# Congenital cataracts in an Ayrshire herd: a herd case report

**DOI:** 10.1186/2046-0481-67-2

**Published:** 2014-01-25

**Authors:** Lea Krump, Luke O’Grady, Ingrid Lorenz, Terence Grimes

**Affiliations:** 1 School of Veterinary Medicine, University College Dublin, Belfield, Dublin 4, Ireland

**Keywords:** Cataracts, Congenital, Bovine, Ophthalmology, Ayrshire, Herd Health

## Abstract

An Ayrshire dairy herd was investigated for occurrence of ocular abnormalities in new-born calves. Ophthalmic examinations were performed on all the animals in the herd and 26% of them were diagnosed with bilateral cataracts. Cataracts varied in extent and severity but the majority were restricted to the lens nucleus. Epidemiological analysis showed the prevalence was higher in male animals and lower in animals born to heifers. A family tree was designed but no genetic impact of dam lines was evident. Sire data was incomplete and could therefore not be included. Based on the information provided by the farmer there was no obvious environmental or nutritional cause of these cataracts. However, data records were incomplete and further investigation/monitoring of the herd would be needed to establish a cause and enable a better insight into the aetiology of this disease in cattle.

## Background

Cattle can be affected by several congenital ocular conditions including cataracts. Congenital cataracts are lens opacities present at birth and expressed in various degrees of severity. They are usually bilateral and confined to the nucleus. There is limited information on this defect in the literature. One of the few comprehensive studies on this disease in cattle was published over 35 years ago and reports a prevalence of up to 34% [1]. However a more recent publication has reported an incidence as high as 79.5% in a herd [2].

The aetiology of this ocular condition is not well established. The development of the lens nucleus occurs between 30 to 60 days of gestation [3] and any insult to the dam which impacts the developing foetus at this stage could contribute to the development of cataracts. Cataracts have been reported in calves exposed to Bovine Viral Diarrhoea (BVD) Virus in utero [4], indicating the importance of infectious diseases as a possible causative agent. Other non-infectious environmental factors such as electromagnetic radiation may also be contributing to the development of congenital cataracts [5]. Nutrition of the cow during pregnancy is crucial for the development of the foetus and deficiencies as well as intoxications at that stage could cause developmental abnormalities. For example, vitamin A deficiency of the dam can contribute to the occurrence of ocular cataracts in calves [6].

Congenital cataracts have been reported as a hereditary condition in several bovine breeds [7-9] but a heritability estimate has not yet been determined. Only recently the genetic mutation responsible for multiple ocular defects (including microphthalmia, persistent hyaloid artery and retinal detachment) in Japanese black cattle has been discovered [10] but congenital cataracts in this breed have not been reported. Congenital cataracts have also never been described in Ayrshire cattle. However, other congenital ocular abnormalities, such as microphthalmia/anophthalmia syndrome [11] and heterochromia of the iris [12] have previously been reported in this breed.

The aim of this paper is to present a herd case of congenital cataracts and to discuss its potential causes. To the best of the authors’ knowledge, this is the first report of congenital cataracts in the Ayrshire breed.

## Case presentation

### Herd description

The herd investigated is a pedigree Ayrshire herd of 110 milking cows in Northern Ireland. The farm extends over 160 acres of grassland. It is a dairy enterprise with an average milk yield of approximately 6000 litres per cow and year operated by the proprietor with one additional farm labourer.

### Farm management

The calving season is spread over the entire year, with the majority of animals calving from September to July. Artificial insemination is performed using several different sires, in addition to two pedigree stock bulls. A new bull is introduced to the farm every year, replacing one of the stock bulls. All breeding animals are vaccinated annually against BVD virus and Leptospirosis since 2005 as well as Bovine Herpes Virus type 1 (Infectious Bovine Rhinotracheitis, IBR) since 2010. The cows are grazed in the summer and housed in a cubicle house during the winter period. Medium Density Fiberboard (MDF) sawdust is used as bedding material in the cubicles. Milking cows are fed grass silage and concentrates according to yield, whereas dry cows are fed straw and silage ad libitum. All cows get mineral supplementation. After an outbreak of abortions and stillbirths (8 out of 25 cows) in the winter of 2011 all the dry cows and pregnant milking cows were fed mycotoxin binders (Micron Ultrasorb™ and Biotal Toxisorb™) as a solution to a suspected fungal contamination of the feed which might have contributed to the problem.

### Clinical history

An ocular problem was first noticed by the farmer in spring 2011 when he reported a calf born without eyes. Since that time he reports of calves born with two different ocular abnormalities; some were born with anophthalmia/microphthalmia (as later diagnosed on post mortem examination) and others with white intra-ocular opacities. In spring 2012 a total of 12 calves were born with visible lens opacities alone, while in autumn of 2012 a further 8 calves were born with cataracts and two with anophthalmia/microphthalmia syndrome. In 2013 a further 12 calves were born with cataracts and one with anophthalmia/microphthalmia. Calves with anophthalmia/microphthalmia were euthanized shortly after birth. According to the farmer calves with cataracts showed no other clinical signs and were thriving well over time. The farmer also expressed the opinion that the dams of the affected calves had no visible ocular problems, and calves with ocular problems were never born to maiden heifers.

### Herd investigation

**Figure 1:**
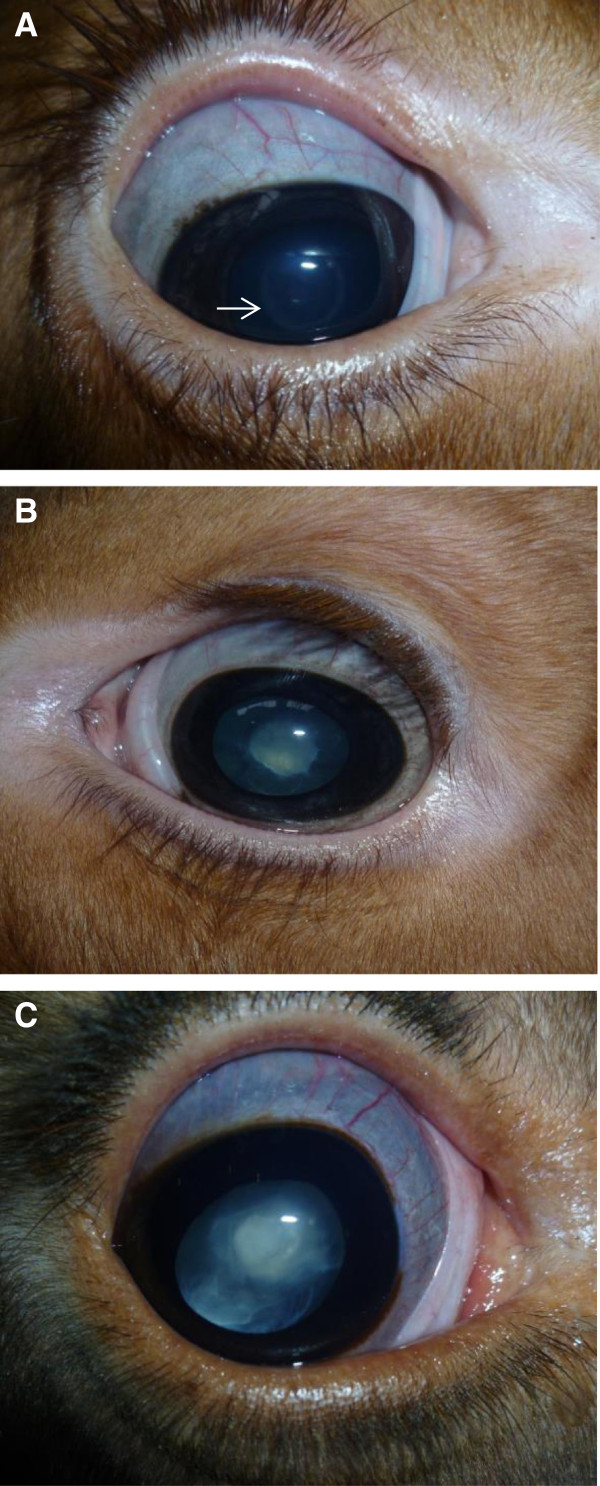
**Photographs of calves with different severity of cataracts. A)** Photograph of a calf with central demarcation (arrow pointing to the ring-like structure) around the nucleus (Group **A**); **B)** Photograph of a calf with a nuclear cataract (Group **B**); **C)** Photograph of a calf with a cataract extending into the perinuclear cortex (Group **C**).

In February 2013 ophthalmic examinations were performed on all animals in the herd (n = 208). Ocular examinations were performed in dim light using a magnifying visor and a Finnoff illuminator. Direct ophthalmoscopy was used for intra-ocular observations. Mydratics were not used but care was taken to obtain oblique views of peripheral lens substance. The cataracts observed were usually bilateral and confined to the nuclear zone. Opacities were mainly rounded in outline but some showed a spiky surface of interface with surrounding clear lens substance. The density of cataracts varied from complete opacity obscuring fundus detail to a barely perceptible zone of central demarcation of a refractive change. Animals were categorised as non-cases or into three groups depending on the appearance of the cataract; group A (Figure 1A) showing central demarcation, group B (Figure 1B) showing opacities confined to the nucleus and group C (Figure 1C) showing cataracts extending into the perinuclear cortex. Three animals had unilateral cataracts, one of which was associated with a persistent hyaloid artery. These three animals, together with calves born with anophthalmia/microphthalmia, were classified as non-cases and excluded from the subsequent analysis. In no animal was a papillary or peri-papillary colobomatous defect identified. In addition, none of the examined animals were showing obvious signs of impaired vision on the day of the investigation. For the purpose of this study, all animals with bilateral cataracts were considered a case. Although it was not certain the cataract was present at birth in all the cases, they were categorised as congenital, based on the appearance and position of the lesion as well as the fact that they were bilateral. Altogether, 54 animals (26%) were identified with bilateral congenital cataracts, of which 39 were classified as group A, 9 as group B and 6 as group C (Table 1).

**Table 1:** Number and sex of animals in different groups based on the extent of the cataract

	**All animals (n)**	**Male (n)**	**Female (n)**
In the herd	208	5	203
With cataracts	54	3	51
Group A	39	1	38
Group B	9	1	8
Group C	6	1	5

Included in the table is the distribution of cases in male and female animals.

An epidemiological investigation of genetic, nutritional and potentially toxic environmental factors was performed based on record analysis and information provided by the farmer. A chi-square test was applied to evaluate the effect of dam and her parity on the occurrence of cataracts. Due to the low number of male animals on the farm, Fischer’s exact test was used to evaluate the effect of sex on the occurrence of cataracts. A non-parametric Kruskal-Wallis test was used to determine if age was associated with severity of lesions.

**Figure 2:**
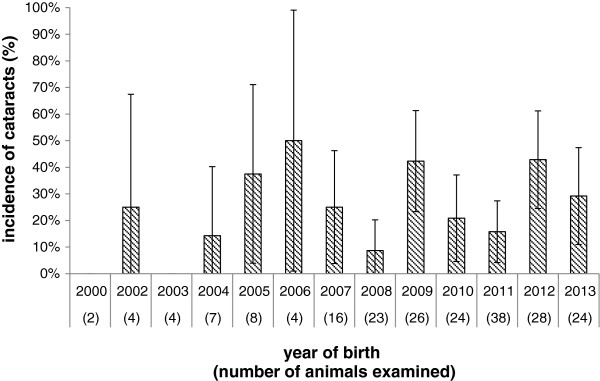
Incidence of congenital cataracts by year of birth with a confidence interval of 95% (represented by error bars).

**Figure 3:**
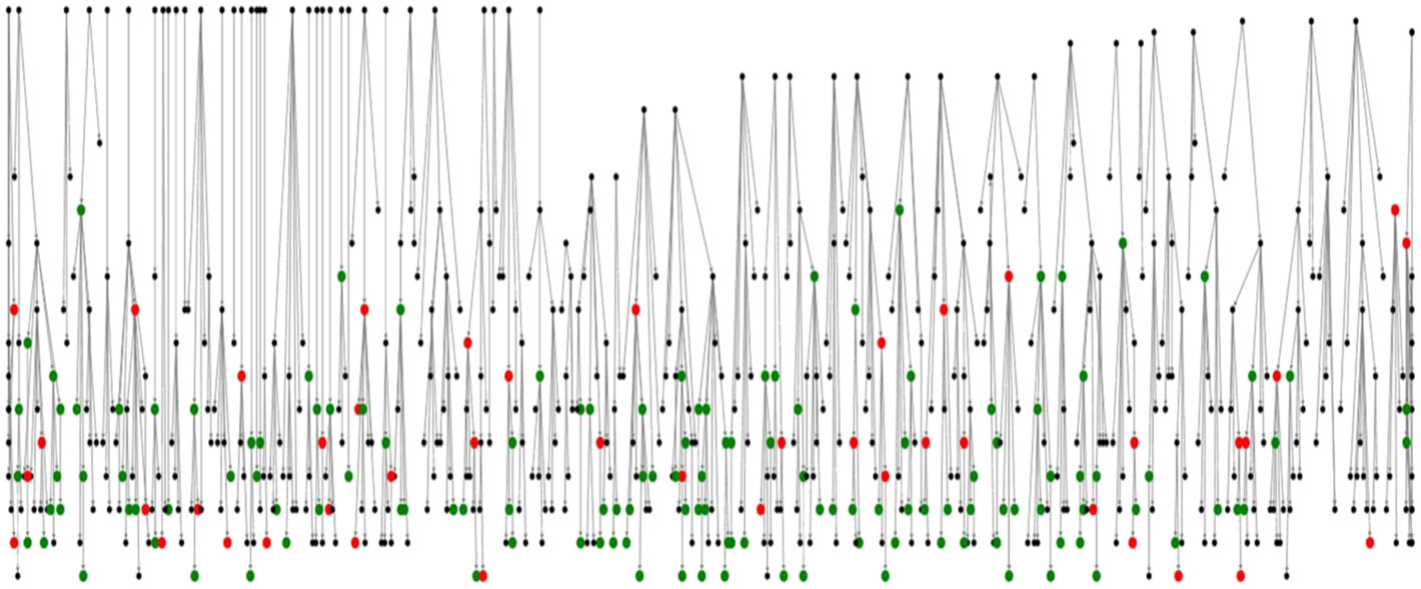
**Family tree (dam line only).** The red dots represent animals with cataracts, the green dots represent animals with no ocular abnormalities and the black dots represent the animals not examined (not in the herd). Data for sires was not available. Animals with cataracts are spread evenly across the diagram, suggesting there is no hereditary component.

The oldest affected animal was born in 2002 and the incidence varied over time (Figure 2). The occurrence of cataracts was evenly distributed over all seasons without any significant difference in month of calves’ births. The average age of animals in group A was 40 months, whereas the average age in group B and C were 31 and 19 months, respectively. The severity of lesions appeared worse in younger animals but this was not statistically significant (p = 0.55). A comparative examination of the effect of dam parity on the occurrence of cataracts indicated that primiparous animals were less likely to produce an affected new-born (p = 0.08), with 17% of cases born to heifers and 29% born to cows. While the majority of the animals available for examination were female (97.6%), the analysis indicated that cataracts were more likely to occur in male animals (p = 0.11). A family tree was used to examine if problem cases were clustered within cow family lines. As no complete sire information was available no evaluation of sire line was possible. Examination of the cow line where animals had been examined shows an even spread of cataracts across the herd (Figure 3). Also, the presence of cataracts in the dam had no effect on the presence of cataracts in their offspring (p = 0.92). As milk recording data was limited, we were unable to investigate the link between milk yield or milk solids and the occurrence of cataracts. On a herd level, a high percentage (≥25%) of early lactation cows (between 1 and 60 days after calving) had milk protein levels below 3% and fat to protein ratios greater than 1.5 in spring 2012. The situation was worse in 2011, when animals had low protein levels and high fat to protein ratios throughout the entire year. These results indicate a potential negative energy balance issue on the farm.

### Previous findings

Prior to this investigation there were post-mortem examinations carried out by the Agri-Food and Biosciences Institute in Omagh (United Kingdom). In May 2012 a calf was diagnosed with a unilateral white opacity in the lens. In January 2013 a calf was diagnosed with anophthalmos and patent ductus arteriosis. In addition, one calf was diagnosed with anophthalmos at University College Dublin (Ireland) in October 2012.

Bulk milk analysis was carried out in November 2012. It was positive for *Fasciola hepatica*, IBR and *L. hardjo* antibodies on ELISA and negative for BVD virus on PCR. Since the beginning of 2013, all calves born are sampled for BVD virus, as a part of national disease eradication programme and until August 2013 all results have been negative. Prior to that, there was no comprehensive investigation of the infectious disease status of the herd carried out. Throughout 2012 a few cows were examined for blood metabolites and minerals without any significant abnormalities detected.

## Discussion

The overall prevalence of bilateral cataracts in this Ayrshire herd was 26%. The oldest animal with this condition was over 10 years of age, even though the farmer had not noticed any eye problems until a case of anophthalmia in 2011. Unlike anophthalmia, mild cataracts can easily be missed with the naked eye. These cataracts are non-progressive. On the contrary, as the lens grows, the effect of the lesion is likely to decrease. That might account for the fact that younger animals appeared more severely affected in comparison to older animals, even though it was not statistically significant. In spite of small number of bulls on this farm, there is a trend showing male animals might be at higher risk for this condition, which is consistent with other reports [13].

There may be several different contributing factors in the development of congenital cataracts. A hereditary cause has been reported in several bovine breeds. In the Holstein-Friesian [7] it is autosomal recessive, whereas in the Jersey it can be autosomal dominant [8] or recessive [14]. Sporadic congenital cataracts have also been reported in the Hereford and Shorthorn [9,15] but never in the Ayrshire breed. However, in this case a pattern of inheritance was not determined but the data was limited and a complete analysis not possible.

Similar limitations also apply to nutritional analysis as comprehensive records were not kept. It is unlikely that nutritional factors were the sole cause of cataracts in this herd, since animals were not showing any other clinical signs of malnutrition. Vitamin A deficiency is known to have a negative impact in cattle on their growth, reproductive performance, immunity and vision. It is important for foetal development and it has recently been reported as a cause of congenital cataract in calves [6]. However, cattle on pasture should consume sufficient β-carotene (Vitamin A precursor) to prevent such problems. Animals in this Ayrshire herd were grazed in the spring and summer and there was no increased incidence of ocular problems in the winter or when cows where housed during the first trimester of gestation, which would be suggestive of vitamin A deficiency on indoor diet. Metabolites like glucose, urea or calcium in the dam do not seem to have an impact on the occurrence of cataracts in calves [16]. A more recent study suggests that decreased antioxidant levels together with increased oxidative stress through lipid mobilisation might contribute to the occurrence of cataracts in cattle [17]. Oxidative stress is considered to be an important mechanism in cataract formation [18] and there is some evidence of negative energy balance and lipid mobilisation in early lactation cows on this farm but a correlation with cataracts could not be established. The status of blood metabolites and minerals for these cows was not measured as a part of this investigation.

Feedstuff additives, such as mycotoxin binders, have not yet been described as a cause of congenital cataracts in cattle. Moreover, these additives have only been used on this farm since 2011, whereas the oldest animal with nuclear lesions was born in 2002 and are hence unlikely to have caused cataracts. The same applies to MDF bedding, which has only been used on this farm since 2009. There are many other environmental factors, which could have contributed to the occurrence and severity of congenital cataracts. Even proximity to mobile telephone masts has been discussed [5] but it is impossible to evaluate all environmental factors during such a herd investigation.

We have investigated some potential infectious agents, which could have caused cataracts in this herd. BVD virus infection in utero can cause malformations of the nervous system as well as ocular cataracts in new-born calves [4]. A complete assessment of this herd’s BVD status has not been conducted. However, given the negative results from previous analysis and lack of any other clinical signs of BVD virus, there is little evidence to date that would suggest BVD on this farm and as such it is unlikely that BVD is causing the ocular problem. Other viral infections such as Akabane virus [19] and Bluetounge virus [20] can cause congenital ocular disorders, but none of them have previously been associated with cataracts, and are not common in Ireland.

Congenital cataracts may be associated with other ocular maldevelopments such as microphthalmia and persistent hyaloid artery [21]. However, we were not able to determine whether there is an association between cataracts and the few cases of anophthalmia/microphthalmia found on this farm.

## Conclusions

In conclusion, no definitive cause of congenital cataracts in this herd was established. On the other hand, none of the mentioned risk factors can be excluded as a potential contributing factor in increasing the severity of these ocular lesions. In order to conduct a more comprehensive investigation in the future, we have advised the farmer to examine every calf born and record results. Also a detailed record of nutritional management together with milk recording data would be beneficial. Information of all the sires used should be available to determine a pattern of heredity. Similarly, examining Ayrshire herds genetically linked to this farm would be useful. A broader investigation to include contiguous herds would allow a better insight into the effects of possible environmental causes, such as electromagnetic sources. Studies suggest that environmental factors are mainly responsible for this disease [1] but it is possible that the cataracts result from an interaction between environmental and genetic factors, as for many other congenital malformations [22]. Even though it has been nearly a century since this condition was first reported in cattle [23], its aetiology remains uncertain.

## Competing interests

The authors declare there are no competing interests.

## Authors’ contributions

LK drafted the manuscript, LOG carried out the epidemiological analysis, IL supervised the production of the manuscript, and TG carried out the ophthalmic examinations. All authors have read and approved the final manuscript.
